# Effect of carboxymethyl cellulose edible coating containing *Zataria multiflora* essential oil and grape seed extract on chemical attributes of rainbow trout meat

**Published:** 2014

**Authors:** Mojtaba Raeisi, Hossein Tajik, Javad Aliakbarlu, Sima Valipour

**Affiliations:** 1 *Department of Public Health, Faculty of Health, Golestan University of Medical Sciences, Gorgan, Iran; *; 2 *Department of Food Hygiene and Quality Control, Faculty of Veterinary Medicine, Urmia University, Urmia, Iran; *; 3 *Graduate Student of Veterinary Medicine, Faculty of Veterinary Medicine, Urmia University, Urmia, Iran.*

**Keywords:** Fish meat, Thiobarbituric acid, Total volatile basic nitrogen

## Abstract

Meat products, especially fish meat, are very susceptible to lipid oxidation and microbial spoilage. In this study, first, gas chromatography mass spectrometry (GC-MS) analysis of *Zataria multiflora* essential oil (ZEO) components was done and then two concentrations of ZEO, (1% and 2%) and two concentrations of grape seed extract (GSE), (0.5% and 1%) were used in carboxymethyl cellulose coating alone and in combination, and their antioxidant effects on rainbow trout meat were evaluated in a 20-day period using thiobarbituric acid reactive substances (TBARS) test. Their effects on total volatile basic nitrogen (TVBN) and pH were evaluated as well. The main components of ZEO are thymol and carvacrol. These components significantly decreased production of thiobarbituric acid (TBA), TVBN and pH level of fish meat. The initial pH, TVBN and TBA content was 6.62, 12.67 mg N per 100 g and 0.19 mg kg^-1^, respectively. In most treatments significant (*p* < 0.05) effects on aforementioned factors was seen during storage at 4 ˚C. The results indicated that use of ZEO and GSE as a natural antioxidant agents was effective in reducing undesirable chemical reactions in storage of fish meat.

## Introduction

In the recent years, use of edible films and coatings for food protection and preservation has increased because of some advantages such as edibility, biocompatibility, being non-toxic, non-polluting and having low cost.^1^^,^^2^ In addition, films and coatings, could act as carriers of food additives like antioxidants and antimicrobials.^3^

Carboxymethyl cellulose is a water-soluble polysaccharide with appropriate biodegradable and edible film-forming properties and can be produced at low cost and large scale from different resources.^4^ It is odorless, tasteless and nontoxic, bears 4 to 5.5% moisture and pH value about 6 to 8.5.

Rainbow trout is one of the fatty farmed fish that has high levels of moisture, nutrient content, and high pH that make it easily perishable product.^5^ The spoilage of fish is usually caused by biological reactions such as oxidation of lipids, microbial growth and metabolic activities. Lipid oxidation is one of the major causes of quality deterioration in fish muscle,^6^ which cause off-orders and off-flavor and reduce the quality and shelf life of the fish during storage time. Thus, keeping good quality and delay in the spoilage of fish meat during storage are major concerns of investigators.^5^^,^^7^ Use of antioxidants such as plant essential oils are appropriate tool to delay the oxidation of lipids and keeping quality of fish meat and thus, increasing shelf life of fish meat during refrigerated storage.^8^^,^^9^

*Zataria multiflora *Boiss (Avishan-e-Shirazi in Persian) is a member of *Labiatae *family that grows in some area of Iran such as central and southern parts^10^ and has various therapeutic effects such as antiseptic, antispasmodic, antiviral, antibacterial and antifungal properties. Thymol and carvacrol, are the main constituents of this essential oil.^10^^,^^11^

Grapes (*Vitisvinifera*) are most popular fruit in the world.^12^ Grape seed extract is rich source of monomeric phenolic compounds, such as catechins, epicatechin and epicatechin-3-O-gallate and dimeric, trimeric and tetrameric procyanidins. These compounds act as anti-mutagenic and antiviral agents.^13^

The aim of this study was to prepare carboxymethyl cellulose coating containing ZEO and GSE and to evaluate their effect on chemical factors related to spoilage of fish meat including lipid oxidation and TVBN production and changes in pH value of fish meat during storage at 4 ˚C.

## Materials and Methods

**Materials. **Carboxymethyl cellulose with average molecular weight of 41,000 g mol^-1^ and GSE were obtained from Sigma, (St. Louis, USA). Polyethylene glycol with average molecular weight of 400 g mol^-1^ and glycerol as plasticizers were obtained from Merck (Darmstadt, Germany).

Fresh aqua-cultured rainbow trout with average weight of 200-300 g were obtained from a cold water aquaculture farm located on Urmia (West Azerbaijan, Iran).

**Gas chromatography mass spectrometry (GC-MS) analysis. **The aerial parts of *Zataria multiflora *was purchased from local grocery store and authenticated at Faculty of Agriculture, Urmia University, Urmia, Iran. Essential oil was obtained using a Clevenger-type collector with hydro-distillation method for 3 hr**. **The essential oil was dehydrated using sodium sulfate and then filtered by 0.22 μm filters. The filtrate was stored in sealed vials at 4 ˚C. The constituents of EO were identified by GC-MS.^14^ The chromatograph was equipped with DB5 capillary column (Length 30 mm, 0.25 mm inner diameter, and 0.25 μm film in thickness). The data were obtained under the following conditions: Initial temperature 50 ˚C, final temperature 250 ˚C and using an ionization energy of 70 eV.

**Preparation of coating solutions and samples. **Coating solutions were prepared dissolving 1 g carboxymethyl cellulose in 100 mL distilled water. Glycerol was added as plasticizer at 0.4 g per 100 mL to carboxymethyl cellulose solution.^4^ Solutions were heated until 85 ^°^C, then kept at this temperature for 5 min. They were then cooled to room temperature and ZEO in two concentrations (1 and 2%) and GSE in two concentrations (0.5% and 1%) were added to coating solutions. The whole fish samples with average weight of 200 to 300 g were randomized into nine groups including the control (untreated) group and eight groups treated with different concentrations of GSE (0.5 and 1%) and ZEO (1%, 2%) alone and in combination (ZEO 1% - GSE 0.5%, ZEO 1% - GSE 1%, ZEO 2% - GSE 0.5%, ZEO 2% - GSE 1%). Immerging method was used for coating the whole Fish samples, and then the samples were allowed to drain in order to form the edible coatings, after that they were stored at 4 ^°^C for chemical analyses performed on days 0, 5, 10, 15, and 20.

**Determination of pH. **To determine pH value, a mixture of 10 g of meat in 100 mL of distilled water was prepared and digital pH meter was used.

**Determination of thiobarbituric acid reactive substances (TBARS). **The thiobarbituric acid index was determined based on the method described by Jeon *et al*. It is based on reaction between thiobarbituric acid with secondary lipid oxidation products such as aldehydes and ketones. The absorbance of TBA is determined at 530 nm and TBA value demonstrated as mg of malonaldehyde equivalents per kg of meat.^15^

**Determination of TVBN. **Determination of TVBN values was based on the method of Pikul *et al*.^16^ The micro-diffusion method was used by distillation after adding MgO to the homogenized samples. TVB-N values were measured with a Kjeldahl type apparatus. Results are expressed in milligram of nitrogen per 100 g of sample.

**Statistical analysis. **All experiments were carried out in triplicate and analysis of variance (ANOVA) in SPSS statistics software (Version 19; SPSS Inc., Chicago, USA) were used for statistical analysis of data. To compare differences between mean values, Duncan's test was used and *p *< 0.05 was considered statistically significant.

## Results

**Identification of volatile components from essential oil. **The results of GC-MS analyses of compounds of ZEO revealed that essential components were carvacrol (18.20%), thymol (13.40%), o-isopropyltoluene (7.54%), linalool (7.40%), 3-methylresacetophenone (4.21%), gamma terpinene (4.11%) and thymol methyl ether (3.55%).

**pH content of fish meat. **Changes in pH value during storage at 4 ˚C for 20 days are shown in Table 1. Initial pH of untreated sample was 6.62. The pH of the untreated fish meat showed higher increase and at the end of the storage period it reached to 7.12. For the treated samples containing ZEO and GSE, pH value was lower than that of untreated sample in most sampling periods (*p* < 0.05). A pH value of treated sample containing 2% essential oil and 1% GSE reached to 6.49 that showed significantly lower pH than untreated sample (*p* < 0.05).

**Thiobarbituric acid reactive substances values of fish meat. **Effect of ZEO and GSE on TBA level are shown in Table 2. Significant increase in TBARS was observed in most sampling periods during storage at 4 ^°^C. In untreated sample, initial TBARS value was 0.19 mg kg^-1^ of meat whereas at the end of the storage period, it reached to 2.05 mg kg^-1^ of meat. In treated samples the increase in TBARS value was significantly lower than that of untreated sample (*p* < 0.05). The highest decrease in lipid oxidation was observed in sample containing 2% essential oil in combination with 1% grape seed extract.

**Level of TVBN in fish meat. **Changes of TVBN values in treated and untreated samples are shown in Table 3. The initial TVBN level for samples were in range between 9 to 13 mg nitrogen per 100 g. For both treated and un-treated samples TVBN value was increased progressively during storage period. The values of TVBN in untreated sample reached to 52 mg nitrogen per 100 g while in treatment groups containing 2% ZEO and 1% GSE, the TVBN value was significantly lower (*p* < 0.05). 

**Table 1:** pH values evaluation in the muscle of rainbow trout during storage at 4 ˚C.

**Groups**	**Day**				
	**0**	**5**	**10**	**15**	**20**
**GSE 0.5%**	6.59 ± 0.10^a^	6.61 ± 0.02^a^	6.63 ± 0.01^a^	6.65 ± 0.10^a^	6.68 ± 0.05^a^
**GSE 1%**	6.57 ± 0.05^a^	6.66 ± 0.07^a^	6.61 ± 0.02^a^	6.59 ± 0.05^b^	6.63 ± 0.01^b^
**ZEO 1%**	6.57 ± 0.10^a^	6.59 ± 0.02^a^	6.61 ± 0.05^a^	6.53 ± 0.10^c^	6.56 ± 0.07^c^
**ZEO 2%**	6.54 ± 0.07^a^	6.57 ± 0.10^b^	6.57 ± 0.01^b^	6.49 ± 0.05^d^	6.52 ± 0.02^d^
**ZEO 1% + GSE 0.5%**	6.56 ± 0.10^a^	6.54 ± 0.01^b^	6.56 ± 0.02^b^	6.51 ± 0.07^d^	6.53 ± 0.05^d^
**ZEO 1% + GSE 1%**	6.55 ± 0.10^a^	6.57 ± 0.02^b^	6.57 ± 0.07^b^	6.49 ± 0.05^d^	6.51 ± 0.01^d^
**ZEO 2% + GSE 0.5%**	6.54 ± 0.02^a^	6.55 ± 0.01^b^	6.58 ± 0.07^b^	6.48 ± 0.07^d^	6.50 ± 0.10^d^
**ZEO 2% +GSE 1%**	6.53 ± 0.02^a^	6.53 ± 0.05^b^	6.55 ± 0.01^b^	6.46 ± 0.02^d^	6.49 ± 0.07^d^
**Control**	6.62 ± 0.02^a^	6.65 ± 0.01^c^	6.63 ± 0.07^a^	6.79 ± 0.05^a^	7.12 ± 0.10^a^

ZEO: *Zataria multiflora* essential oil; GSE: Grape seed extract.

abcd Different letters in the same column indicate significant differences (*p* < 0.05).

**Table 2:** Thiobarbituric acid reactive substances values in muscle (mg kg^-1^ of meat) of rainbow trout during storage at 4 ˚C

**Groups**	**Day**				
	**0**	**5**	**10**	**15**	**20**
**GSE 0.5%**	0.21 ± 0.01^b^	0.37 ± 0.03^b^	0.92 ± 0.01^b^	1.07 ± 0.03^b^	1.24 ± 0.03^b^
**GSE 1%**	0.19 ± 0.00^a^	0.30 ± 0.21^c^	0.68 ± 0.03^c^	1.04 ± 0.02^b^	1.15 ± 0.02^c^
**ZEO 1%**	0.19 ± 0.01^a^	0.24 ± 0.01^d^	0.54 ± 0.01^d^	0.59 ± 0.01^c^	0.95 ± 0.01^d^
**ZEO 2%**	0.16 ± 0.00^a^	0.23 ± 0.00^d^	0.33 ± 0.01^e^	0.42 ± 0.01^d^	0.70 ± 0.00^e^
**ZEO 1% + GSE 0.5%**	0.18 ± 0.00^a^	0.24 ± 0.00^d^	0.49 ± 0.00^f^	0.52 ± 0.00^e^	0.84 ± 0.02^f^
**ZEO 1% + GSE 1%**	0.18 ± 0.00^a^	0.25 ± 0.02^e^	0.53 ± 0.00^g^	0.56 ± 0.02^e^	0.91 ± 0.03^f^
**ZEO 2% + GSE 0.5%**	0.17 ± 0.00^a^	0.23 ± 0.00^d^	0.34 ± 0.00^h^	0.42 ± 0.01^d^	0.60 ± 0.00^g^
**ZEO 2% +GSE 1%**	0.16 ± 0.00^c^	0.21 ± 0.00^d^	0.29 ± 0.00^h^	0.44 ± 0.02^d^	0.55 ± 0.00^g^
**Control**	0.19 ± 0.01^a^	0.44 ± 0.03^a^	1.20 ± 0.01^a^	1.59 ± 0.02^a^	2.05 ± 0.04^a^

ZEO: *Zataria multiflora* essential oil; GSE: Grape seed extract.

**abcdefgh:** Different letters in the same column indicate significant differences (*p* < 0.05).

**Table 3:** Total volatile basic nitrogen values in muscle (mg N per 100 g) of rainbow trout during storage at 4 ˚C

**Groups**	**Day**				
	**0**	**5**	**10**	**15**	**20**
**GSE 0.5%**	11.34 ± 0.58^a^	15.33 ± 0.58^a^	23.33 ± 0.58^a^	32.33 ± 0.58^a^	40.30 ± 0.58^a^
**GSE 1%**	11.33 ± 0.58^a^	14.33 ± 0.58^b^	21.67 ± 0.58^b^	29.67 ± 0.58^a^	37.57 ± 0.58^b^
**ZEO 1%**	10.33 ± 0.58^b^	13.67 ± 0.58^b^	18.33 ± 0.58^c^	28.67 ± 0.58^a^	35.33 ± 0.58^c^
**ZEO 2%**	9.33 ± 0.58^c^	12.67 ± 0.58^c^	15.33 ± 1.15^d^	23.00 ± 1.00^b^	31.60 ± 1.15^d^
**ZEO 1% + GSE 0.5%**	10.33 ± 0.58^a^	13.33 ± 1.15^b^	17.33 ± 1.53^b^	22.33 ± 1.15^d^	33.33 ± 0.58^d^
**ZEO 1% + GSE 1%**	9.67 ± 0.58^b^	12.67 ± 0.58^c^	18.00 ± 1.00^e^	23.00 ± 1.00^b^	34.33 ± 0.58^f^
**ZEO 2% + GSE 0.5%**	9.33 ± 1.15^c^	12.33 ± 1.15^b^	15.33 ± 0.73^d^	21.33 ± 1.00^b^	31.00 ± 1.00^d^
**ZEO 2% +GSE 1%**	8.67 ± 0.58^c^	12.00 ± 1.00^c^	14.00 ± 2.00^d^	20.67 ± 3.79^c^	26.30 ± 0.58^g^
**Control**	12.67 ± 0.58^a^	17.33 ± 0.58^c^	25.33 ± 0.58^a^	36.33 ± 0.58^a^	52.00 ± 1.00^a^

ZEO: *Zataria multiflora* essential oil; GSE: Grape seed extract.

**abcdefg:** Different letters in the same column indicate significant differences (*p* < 0.05).

## Discussion

Results obtained from GC-MS analyses of ZEO compounds, showed that EO is rich in thymol and carvacrol which have suitable antibacterial and anti-oxidant properties. Among the phenolic compounds, carvacrol has been shown to have the highest anti-microbial activity due to its hydrophobic nature and the presence of a free hydroxyl group which is essential for its activity on cell membranes.^11^

Fish have little carbohydrate in their tissue and thus a little lactic acid is produced in their tissue.^17^ pH value was increased during storage of meat in cold condition. Most important reason asserted by investigators is increase in total volatile basic nitrogen that is produced by endogenous or bacterial enzymes.^18^^,^^19^ The pH value in fish meat is affected by post mortem changes and environmental conditions like seasons and stress during the catch and diet.^20^ Changes in pH value can be used as an indicator for determining freshness of fishery products.^17^ Increase in pH value of fish muscle has undesirable effects on sensorial characteristics and consequently decreases shelf life of fish meat.^17^^,^^18^ The pH of treated samples containing essential oil and GSE were consistently lower than untreated sample during cold storage, which might relate to antibacterial effect of ZEO and grape seed extract. The results of this study are in accordance with the results of Mohan *et al*. who found that sardine fillets treated with chitosan coating showed lower pH and TBA compared to the control treatments.^20^ Many researchers demonstrated suitable effects of coatings on reducing pH value in different products.^19^^,^^21^^,^^22^

Thiobarbituric acid value is assumed as an indicator of lipid oxidation.^23^ Oxidation of unsaturated fatty acids might increase TBA level and increase in TBA level lead to raise in bitterness of fish meat and consequently causes undesirable effect on quality characteristics of fishery products.^24^ The results obtained from this study showed that lipid oxidation in fish meat was reduced when coating was enriched with ZEO and grape seed extract. This reduction in lipid oxidation is due to the antioxidant effect of essential oil and GSE and also reduction permeability of oxygen to fish lipids. Results obtained from current study is in agreement with other studies.^23^^-^^25^ Ahmad *et al.* suggested that sea bass slices wrapped with gelatin film incorporated with lemongrass essential oil produced less TBA than control group.^19^

Production of TVBN is one of the most important reasons for spoilage of fish meat.^26^ During storage of fish meat, activity of some special proteolytic bacteria lead to degradation of non-protein nitrogen compounds and proteins.^26^^,^^27^ Accumulation of total volatile basic nitrogen has undesirable effect on sensorial properties of fish meat specially smell of fishery products.^27^ Results of this study showed that coating enriched with ZEO and GSE has appropriate effect on decrease in production of total volatile basic nitrogen. Ojagh* et al. *reported similar results in their study on effect of chitosan coating on quality properties of rainbow trout meat.^28^

In conclusion, carboxymethyl cellulose edible coating enriched with ZEO and GSE has desirable potential to preserve rainbow trout meat and could retard chemical reactions related to spoilage of fish meat during refrigerated storage. 
